# The Reverse Distance Effect in Ordinal Processing of Repeated Item Sequences

**DOI:** 10.3390/bs16040582

**Published:** 2026-04-13

**Authors:** Zhengping Huang, Rui Fu, Hua He

**Affiliations:** 1 Department of Psychology, School of Education, Soochow University, Suzhou 215123, China; 20234218046@stu.suda.edu.cn (Z.H.); fr2630978777@163.com (R.F.); 2 Department of Psychology, School of Social and Behavioral Sciences, Nanjing University, Nanjing 210023, China

**Keywords:** ordinal processing, reverse distance effect, repeated item sequences, number, letter

## Abstract

Ordinal processing is a core component of numerical cognition, and its behavioral signature is the Reverse Distance Effect (RDE). However, previous studies indicate that the RDE is influenced by sequence composition, and it is unclear whether it is similarly affected by sequence types. Prior research has largely focused on non-repeating sequences (e.g., 1-2-3), while the processing of repeated item sequences (i.e., non-strict sequences; e.g., 1-2-2-3) remains unexplored. Experiment 1 (numbers) showed a significant RDE in the first and last repetition conditions. However, the middle repetition condition resulted in a null effect, suggesting that the middle repeated item interferes differentially with ordinal representation. In contrast, Experiment 2 (letters) showed a significant RDE regardless of repetition position. Overall, the RDE extends to repeated item sequences when interference is comparable between distance conditions. Moreover, the presence of the RDE in the letter task indicates that the effect observed in repeated item sequences extends to non-numerical materials. This study provides the first systematic investigation of repeated item sequences, highlighting the role of repeated items in order judgment and expanding the scope of ordinal processing research.

## 1. Introduction

Numerical processing ability is not only closely related to individuals’ academic achievement but is also a significant predictor of future socioeconomic status (Duncan et al., 2007; Ritchie & Bates, 2013). Numbers are characterized by two fundamental properties: cardinality and ordinality. Cardinality refers to the quantitative information a number conveys, such as the quantity of objects in a set being two. Ordinality refers to the rank or relative position of a number within a defined sequence, such as two is positioned after one and before three (Lyons et al., 2016). Recently, ordinal representations have gained increasing attention. Extensive research indicates that ordinal processing is distinct from cardinal processing and is a stronger predictor of mathematical ability (Goffin & Ansari, 2016; Lyons et al., 2014; Lyons & Beilock, 2013).

Based on the characteristics of their constituent elements, numerical sequences can be categorized into non-repeating and repeated item sequences. Mathematically, the former corresponds to strict sequences, while the latter corresponds to non-strict sequences. Here, we use the ascending order as an example. A strictly ascending sequence requires each term to be greater than the preceding one, and equal adjacent elements are not allowed. Formally, for all *n* ∈ N, if a*_n_*_+1_ > a*_n_*, the sequence is strictly increasing, such as 1-2-3. A non-strictly increasing sequence allows for equality between adjacent elements. Formally, for all *n* ∈ N, if a*_n_*_+1_ ≥ a*_n_*, the sequence is non-strictly increasing, such as 1-1-2-3 (Rosen & Krithivasan, 2013). In this study, we created non-strict sequences by inserting a single adjacent repeated item into strict sequences. For clarity, such sequences are hereafter called repeated item sequences.

Sequences containing repeated elements are common, ranging from mathematics education (e.g., number sequence reasoning) to practical applications in daily life (e.g., tied rankings). Furthermore, given that the Reverse Distance Effect (RDE)—the behavioral signature of ordinal processing (Sella et al., 2020)—exhibits instability across different task and material conditions, its generalizability to different sequence types remains to be verified. From a theoretical perspective, the current study not only reveals how individuals process order information within repeated item sequences but also helps to clarify the boundary conditions of ordinal processing mechanisms. From a practical perspective, this study deepens the understanding of complex sequence processing and provides a basis for sequence learning and instructional interventions in educational settings; it also offers a reference for the assessment and intervention of individuals with order processing deficits.

Ordinal processing is commonly measured using the order judgment task. In this task, participants are required to judge whether a triplet of digits, presented simultaneously in a horizontal array, are arranged in ascending order (e.g., 1-2-3) or not (e.g., 1-3-2). The RDE is typically observed in ordered sequences, where individuals respond faster and more accurately to numerically small distance sequences (e.g., 1-2-3) compared to large distance sequences (e.g., 1-4-7) (Franklin et al., 2009; Sella et al., 2020; Turconi et al., 2006). The RDE contrasts with the canonical distance effect (CDE): when comparing the magnitude of two numbers, pairs with larger numerical distances (e.g., 1 vs. 9) typically elicit shorter reaction times and higher accuracy compared to pairs with smaller distances (e.g., 1 vs. 2) (Moyer & Landauer, 1967).

Two primary theoretical accounts have been proposed to explain the RDE: the Item Association Theory and the Serial Scanning Theory. The Item Association Theory proposed that consecutive sequences (e.g., 1-2-3) have a significantly higher co-occurrence frequency in daily life than non-consecutive sequences (e.g., 1-3-5), resulting in stronger associative links between adjacent numbers (Sasanguie et al., 2017).

During order judgment, consecutive sequences automatically activate these inter-item associations stored in long-term memory, where each item in the sequence serves as a trigger for the consecutive item (Serra & Nairne, 2000). For instance, “2” triggers “1” and “3”—thereby facilitating the order judgment process. In contrast, non-consecutive sequences (e.g., 1-3-5) lack such strong associative support. Consequently, individuals may rely on stepwise magnitude comparison (e.g., 1 < 3 and 3 < 5) or verbal rehearsal (e.g., 1 … 3 … 5 …) to determine the order, resulting in shorter reaction times for consecutive sequences compared to non-consecutive ones (Gattas et al., 2021; Vos et al., 2017). Dubinkina et al. (2021) provided more direct evidence for the use of multiple strategies through a self-report paradigm, finding that individuals employed at least three distinct strategies in ordinal judgments, including memory retrieval and triplet decomposition (i.e., magnitude comparison).

Serial Scanning Theory proposed that the representation of ordinal information relies on a spatially organized mental number line. During the order judgment task, input numerical symbols are mapped onto this spatial representation, and individuals determine the ordinal relationship through the serial scanning of the represented positions. According to this theory, small distance sequences (e.g., 19-20-21) occupy a shorter spatial span on the mental number line, requiring less time to scan, thus leading to the generation of the RDE (Franklin et al., 2009; Wong et al., 2022).

Taken together, current perspectives tend to support a hybrid mechanism: high-familiarity sequences (e.g., single-digit sequences) are processed primarily via item associations, whereas low-familiarity sequences (e.g., two-digit sequences) rely more heavily on serial scanning (Franklin et al., 2009; Vos et al., 2017; Wong et al., 2022).

Repeated item sequences have received extensive attention in cognitive psychology. In sequence processing, researchers have identified the classic Ranschburg effect in immediate serial recall tasks: when a short sequence contains repeated items, the recall accuracy for the second occurrence of a repeated item tends to decline significantly, particularly when the repetitions are separated by intervening items (Jahnke, 1969; Ranschburg, 1905). Conversely, when the two occurrences are adjacent, a facilitation effect is observed for both the repeated positions and overall recall (Crowder, 1968). Furthermore, the Ranschburg effect persists even in tactile stimuli (Johnson et al., 2019), suggesting that the influence of repeated items reflects a deep, intrinsic human processing mechanism.

Crucially, the Ranschburg effect is modulated by the serial position of the repeated items. Crowder (1968) demonstrated that when repeated items appear at the terminal positions of a sequence (i.e., the beginning or end), they elicit neither inhibition nor facilitation. Similarly, Johnson et al. (2019) observed that in six-item sequences, the inhibitory effect was specific to serial position 5, and was not observed at positions 1 or 6. Given that ordinal processing is fundamentally defined by the processing of sequential position, we consider the position of repetition to be a critical variable affecting cognitive processing.

Focusing on numerical cognition, repeated item sequences have important theoretical implications. Existing research has largely focused on cardinal processing, where evidence suggests that repeated and non-repeated sequences may rely on different processing mechanisms. Cardinal processing studies typically employ a same–different task in which two digits (e.g., 1-1, 1-2) are presented simultaneously on the left and right sides of the screen, and participants judge whether they are identical (Dehaene & Akhavein, 1995). The findings show that a numerical size effect can still be observed in repeated digit pairs—for example, responses to 1-1 are slower than to 9-9. The numerical size effect refers to the phenomenon whereby reaction times and error rates increase as numerical magnitude increases (Moyer & Landauer, 1967). Subsequent work suggests that this effect may stem from physical similarity between digits; when the stimulus set was changed to 1, 2, 7, and 8, the size effect in repeated pairs disappeared (Verguts & Van Opstal, 2005, Experiment 2; see also van Opstal & Verguts, 2011). Wong et al. (2018) also found that different physical symbol formats can influence performance in same–different tasks. Together, these findings indicate that in cardinal processing, repeated sequences differ from non-repeated sequences and may rely more heavily on rapid perceptual matching mechanisms at a physical level. Importantly, in the domain of ordinal processing, it remains unclear whether repeated item sequences rely on mechanisms that differ from those involved in non-repeated sequences. More specifically, it is still unknown whether a reversed distance effect emerges in repeated item sequences and whether the serial position of repetitions modulates the underlying cognitive processes.

Research on ordinal processing has expanded beyond the numerical domain. Analogous RDE patterns have been observed in the processing of letter (Vogel et al., 2017; Vos et al., 2017) and month sequences (Morsanyi et al., 2017). However, it remains unclear whether the RDE in repeated item sequences also extends to non-numerical domains.

Vos et al. (2017) observed that letter sequences can also elicit the RDE. Furthermore, they found that numerical processing did not account for additional unique variance in predicting mathematical ability over and above letter processing, suggesting that the RDE reflects a domain-general mechanism. However, Vogel et al. (2017) examined the stability of the RDE across two measurement sessions and found that, although the RDE was observed in both numerical and letter tasks, the RDE in the numerical task demonstrated high test–retest reliability, whereas the reliability for the letter task was significantly lower. Crucially, their results showed that numerical processing did account for unique variance in predicting mathematical ability over and above letter processing. Additionally, in a two-item order judgment task, Sasanguie et al. (2017) and Vogel et al. (2015) found that the letter condition elicited an RDE, whereas the numerical condition exhibited a canonical distance effect. This dissociation suggests that the two domains may rely on distinct processing pathways. Vogel et al. (2017) proposed that because the frequency of ordinal information for letters in daily life is significantly lower than numbers, participants may have adopted inconsistent strategies when judging letter sequences. In summary, the RDE is also commonly observed in non-numerical materials in non-repeating sequences; however, whether it reflects domain-general or domain-specific mechanisms remains controversial.

### Current Study

Although ordinal processing has received increasing attention, the processing of order information within repeated numerical sequences remains unclear. In addition, given that ordinal processing is inherently grounded in positional information, it remains largely unknown how the specific position of repeated items modulates the processing mechanism. Furthermore, whether the Reverse Distance Effect persists across different sequence types remains to be investigated. To address these issues, the present study employed an order verification task to investigate the ordinal processing of repeated numerical sequences (specifically, repeated quadruplets, e.g., 1-2-2-3). Additionally, Experiment 2 utilized repeated letter sequences to examine whether the RDE extends to non-numerical domains.

Based on these theoretical frameworks, we formulated four hypotheses regarding the RDE in ordered sequences:

(a) Item Association Account. This account proposes that the RDE arises from inter-item associative strength. Although repetition disrupts the global associative chain, local associations are partially preserved, and the degree of disruption varies with the repetition position. Consequently, the RDE is expected to vary across repetition positions, predicting an interaction between distance and the repetition position: stronger interference should reduce or eliminate the RDE, whereas weaker interference should preserve it. Such an interaction would support this account, whereas its absence would challenge it.

(b) The Serial Scanning Account. According to this account, the RDE reflects differences in spatial distance between items on the mental number line. Because repeated numbers do not alter their spatial representations, repetition should not influence the scanning process (Devlin et al., 2024; Franklin et al., 2009). Accordingly, this account predicts a robust RDE and no interaction between the distance and repetition position. Any modulation of the RDE by the repetition position would therefore be inconsistent with this account.

(c) The Magnitude Comparison Account. This account suggests that repeated numbers disrupt ordinal processing, leading participants to rely on magnitude comparison strategies. As a result, it predicts the main effect of distance as being consistent with a canonical distance effect, rather than an RDE. The observation of an RDE would therefore contradict this account (Vos et al., 2021).

(d) The Distinct Processing Hypothesis. This hypothesis proposes that repeated item sequences engage a qualitatively different processing mechanism that is independent of both ordinal and magnitude-based processing. Therefore, it predicts no main effect of distance and no interaction involving distance. Any distance-related effect (RDE or CDE) would be inconsistent with this hypothesis.

Table 1 provides a concise summary of the predicted outcomes of the four theoretical accounts.

Regarding the impact of repeated items: if repeated items are simply ignored during processing, reaction times should be independent of the repetition position, regardless of whether the sequence is ascending or mixed. In contrast, if the locus of repetition plays a role in ordinal processing, reaction times should exhibit significant variability depending on the repetition position.

The current study comprises two experiments designed to investigate the cognitive mechanisms underlying the ordinal processing of repeated item sequences. Experiment 1 employed a numerical order judgment task. By using repeated numbers to construct four-item sequences, we examined whether the RDE persists under these conditions and tested the modulating effect of repetition position. Experiment 2 extended this investigation to the letter domain, aiming to verify whether the RDE generalizes to four-letter sequences with repetitions and to explore the influence of repetition position. By systematically examining ordinal processing in repeated item sequences, this study aims to further illuminate the mechanisms underlying the RDE and to examine whether the RDE in non-strict sequences generalizes across domains.

## 2. Experiment 1

### 2.1. Method

#### 2.1.1. Participants

An a priori power analysis was conducted using G*Power 3.1.9.2 for a 2 (order: ascending; mixed) × 2 (distance: small; large) × 3 (repetition position: first, middle, and last) repeated measures ANOVA. The result indicated that a minimum sample size of 21 participants was required (*α* = 0.05, effect size *f* = 0.23, power = 0.95). The effect size parameter was derived from previously reported effect sizes for the Reverse Distance Effect (Gattas et al., 2021; Vogel et al., 2021; Vos et al., 2017).

Thirty-one undergraduate students from a university in China (23 females; *M* = 21.97 years, and *SD* = 1.78) participated in Experiment 1. All the participants were healthy, right-handed, and had normal or corrected-to-normal vision. The study was approved by the local ethics committee, and written informed consent was obtained from all participants prior to the experiment. Participants received monetary compensation after the experiment.

#### 2.1.2. Procedure

Experiment 1 was performed in a sound-attenuated chamber. The experiment was programmed using PsychoPy software (Version 2022.1.2; Peirce et al., 2019). Visual stimuli were displayed on a 27-inch LG monitor (resolution: 2560 × 1440 pixels; refresh rate: 120 Hz). The viewing distance was approximately 60 cm.

#### 2.1.3. Numerical Order Judgment Task

The experimental paradigm and stimuli were adapted from Gattas et al. (2021). We modified the original triplets by introducing repeated items to create numerical quadruplets. For simplicity, all ordered sequences were ascending. On each trial, four numbers were presented simultaneously at the center of the screen. Participants were instructed to judge whether the numbers were arranged in an ascending (e.g., 1-2-2-3) or mixed order (e.g., 1-3-2-2). Responses were made using the ‘A’ and ‘L’ keys on a standard QWERTY keyboard; the response-to-key mapping was counterbalanced across participants.

The numbers ranged from one to nine. The sequences were categorized into ascending and mixed orders. Mixed sequences were generated by rearranging the positions of the numbers from the corresponding ascending sequences. All sequences contained two adjacent repeated digits. This repetition position varied across three levels: first, middle, and last. Following Goffin and Ansari (2016) and Vogel et al. (2021), numerical distance was divided into a small distance condition (inter-item distance = 1; total distance = 2) and a large distance condition (inter-item distance = 2 or 3; total distance = 4 or 6). Theoretically, this classification is based on the view that Distance 1 (consecutive sequences) is processed via memory retrieval, whereas Distances 2 and 3 (non-consecutive sequences) rely on magnitude comparison; this classification is a standard paradigm in current order judgment tasks (Vogel et al., 2021). This classification was applied identically to repeated item sequences, which differed from non-repeated sequences only by the inclusion of the repetition. For the complete experimental stimuli, see the Supplementary Materials.

**Figure 1:**
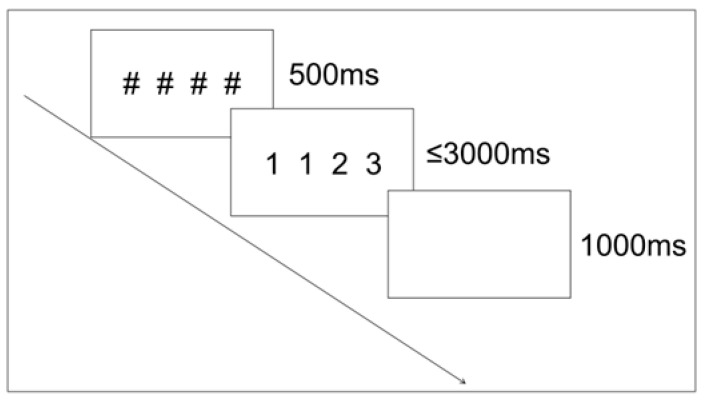
Examples of trial sequences for the numerical order judgment task. The “####” symbol represents the central fixation point.

Stimuli were presented in white Times New Roman font (RGB: 255, 255, 255) on a gray background (RGB: 128, 128, 128), with two spaces between numbers. A typical trial started with a 500 ms fixation (“####”), followed by the target digits (6.9° × 1.4° of visual angle) for a maximum of 3000 ms. The trial ended with a response or upon timeout, followed by a 1000 ms blank screen. See Figure 1 for a schematic of the trial sequence.

Prior to the formal experiment, participants performed 24 practice trials with feedback. No feedback was given during the formal experiment. The formal experiment comprised 420 trials, balanced across conditions and presented in a pseudo-random order, with repetition positions evenly distributed and no more than three consecutive trials sharing the same repetition position. The session was organized into five blocks (84 trials per block), separated by short breaks.

### 2.2. Results

The overall accuracy for the order judgment task approached a ceiling effect (*M* = 97.05%; *SD* = 2.26%). Given the ceiling effect in accuracy and the absence of a significant speed–accuracy trade-off (Pearson correlations: *r* = 0.092; *p* = 0.621), error trials (2.95%) and outlier trials (1.38%; defined as > 3 SD from the mean) were excluded from the analysis. After excluding extreme values, the reaction time data still exhibited moderate-to-high positive skewness (skewness = 1.16) and leptokurtosis (kurtosis = 4.41). Following previous research (Devlin et al., 2024; Vogel et al., 2021; Vos et al., 2017), the median reaction time was used as the statistical measure. Table 2 presents the mean RTs and standard deviations for each condition in Experiment 1.

A 2 × 2 × 3 repeated measures ANOVA was conducted on median reaction times, with Order (ascending vs. mixed), Distance (small vs. large), and Repetition Position (first, middle, and last) as within-subject factors. Post hoc pairwise comparisons were computed using estimated marginal means derived from the fitted model, with the *p*-values adjusted using the Tukey method. Analyses for all two experiments were conducted using the statistical programming language R (Version 4.3.2) within RStudio (Version 2025.09.1+401) (R Core Team, 2023; RStudio Team, 2022).

The ANOVA results revealed a significant main effect of Order, *F*(1, 30) = 36.60, *p* < 0.001, *η_p_*^2^ = 0.55. Specifically, responses were significantly faster in the ascending condition (*M* = 634 ms; *SD* = 127 ms) compared to the mixed condition (*M* = 684 ms; *SD* = 138 ms). The main effect of Distance was also significant, *F*(1, 30) = 23.32, *p* < 0.001, *η_p_*^2^ = 0.44. The responses were significantly faster in the large distance condition (*M* = 649 ms; *SD* = 130 ms) than in the small distance condition (*M* = 669 ms; *SD* = 140 ms). The main effect of Repetition Position was also significant: *F*(2, 60) = 13.61, *p* < 0.001, and *η_p_*^2^ = 0.31. Post hoc comparisons indicated that responses in the middle position (*M* = 672 ms; *SD* = 139 ms) were significantly slower than in the first (*M* = 658 ms and *SD* = 136 ms; *p* = 0.021) and last (*M* = 648 ms and *SD* = 130 ms; *p* < 0.001) positions. No significant difference was found between the first and last positions (*p* = 0.148).

Regarding interactions, there were significant two-way interactions between Order and Distance, *F*(1, 30) = 75.96, *p* < 0.001, and *η_p_*^2^ = 0.72, and between Order and Repetition Position, *F*(2, 60) = 14.33, *p* < 0.001, and *η_p_*^2^ = 0.31. The interaction between Distance and Repetition Position was not significant: *F*(2, 60) = 0.72, *p* = 0.491, *η_p_*^2^ = 0.02. Importantly, the three-way interaction among Order, Distance, and Repetition Position was significant, with *F*(2, 60) = 3.94, *p* = 0.032, and *η_p_*^2^ = 0.12. To decompose the significant three-way interaction, we conducted separate repeated measures ANOVAs on Distance and Repetition Position for the ascending and mixed conditions.

In the ascending condition, the two-way repeated measures ANOVA revealed a significant main effect of Distance: *F*(1, 30) = 5.04, *p* = 0.032, *η_p_*^2^ = 0.14. Specifically, response times were faster for small distance sequences (*M* = 627 ms; *SD* = 127 ms) than for large distance sequences (*M* = 641 ms; *SD* = 129 ms). The main effect of Repetition Position was also significant, with *F*(2, 60) = 12.85, *p* < 0.001, and *η_p_*^2^ = 0.30. Post hoc comparisons indicated that response times at the Last position (*M* = 617 ms; *SD* = 120 ms) were significantly faster than at the First position (*M* = 646 ms, *SD* = 135 ms, and *p* < 0.001) and the Middle position (*M* = 638 ms, *SD* = 127 ms, and *p* = 0.002). The difference between the First and Middle positions was not significant (*p* = 0.261). Importantly, the interaction between Distance and Repetition Position was significant, *F*(2, 60) = 4.73, *p* = 0.034, *η_p_*^2^ = 0.14.

To further interpret this significant interaction, the simple main effects of Distance were examined within each Repetition Position. The results revealed that significant distance effects were observed at both the First and Last positions. Specifically, the effect of Distance was significant at the First position, with *F*(1, 30) = 5.86, *p* = 0.022, and *η_p_*^2^ = 0.16, and at the Last position, with *F*(1, 30) = 9.01, *p* = 0.005, and *η_p_*^2^ = 0.23. In both cases, response times were significantly faster for small distance sequences compared to large distance sequences. In contrast, the distance effect was not significant at the Middle position: *F*(1, 30) = 0.24, *p* = 0.628, *η_p_*^2^ = 0.01. To quantify evidence for the null effect, a Bayesian analysis (default JZS prior; *r* = 0.707) was conducted; the resulting Bayes factor favored the null hypothesis (*BF*_01_ = 4.67), providing moderate evidence for the absence of the RDE in the middle repetition condition. Specific response times and standard deviations are presented in Table 2.

Given the theoretical importance of repetition position outlined in the introduction, the simple main effects of the repetition position were examined separately at each level of distance. For small distance sequences, the main effect of Position was significant: *F*(2, 30) = 8.30, *p* = 0.001, and *η_p_*^2^ = 0.36. Post hoc comparisons revealed that response times at the Last position were significantly faster than at the First (*p* = 0.005) and Middle (*p* = 0.001) positions. There was no significant difference between the First and Middle positions (*p* = 0.789). The overall pattern can be summarized as First = Middle > Last. For large distance sequences, the main effect of Position was also significant, with *F*(2, 30) = 7.29, *p* = 0.003, and *η_p_*^2^ = 0.33. However, the pattern differed: response times at the First position were significantly slower than at the Middle (*p* = 0.045) and Last (*p* = 0.002) positions. No significant difference was found between the Middle and Last positions (*p* = 0.550). The overall pattern can be summarized as First > Middle = Last. Specific response times and standard deviations are presented in Table 2.

In the mixed condition, the simple two-way repeated measures ANOVA revealed a significant main effect of Distance: *F*(1, 30) = 97.54, *p* < 0.001, and *η_p_*^2^ = 0.77. Large distance sequences (*M* = 657 ms; *SD* = 131 ms) were significantly faster than for small distance sequences (*M* = 712 ms; *SD* = 140 ms). The main effect of Repetition Position was also significant, with *F*(2, 60) = 14.78, *p* < 0.001, and *η_p_*^2^ = 0.33. Post hoc comparisons indicated that response times at First position (*M* = 706 ms; *SD* = 142 ms) were significantly slower than at the Last (*M* = 669 ms, *SD* = 136 ms, and *p* = 0.001) and Middle (*M* = 678 ms, *SD* = 133 ms, and *p* < 0.001) positions. The difference between the Last and Middle positions was not significant (*p* = 0.314). However, the interaction between Distance and Repetition Position was not significant, *F*(2, 60) = 1.93, *p* = 0.154, and *η_p_*^2^ = 0.06. Therefore, no further follow-up analyses of this interaction were conducted in the mixed condition.

### 2.3. Discussion

Experiment 1 aimed to examine the RDE in repeated-number sequences and to determine the influence of repetition position. We first described the overall RDE pattern. Next, based on the interaction between distance and repetition position, we proposed an interference account within the Item Association Theory framework. Finally, we addressed the canonical distance effect observed in mixed sequences.

First, regarding ascending sequences, the RDE persisted in the first and last repetition conditions but disappeared in the middle repetition condition. Second, for ascending sequences, the effect of repetition position depended on distance. Small distance sequences showed a “last fastest” pattern (First = Middle > Last), whereas large distance sequences exhibited a “first slowest” pattern (First > Middle = Last). These diverging patterns suggest distinct underlying mechanisms.

Small distance processing relies on inter-item associations. When the repetition is at the last position (e.g., 1-2-3-3), the associative links within the core sequence (1-2-3) remain intact, allowing for fluid processing and faster reaction times. In contrast, repetitions at the first or middle positions disrupt these associations, resulting in slower responses.

Large distance processing is often attributed to either magnitude comparison (Vos et al., 2021) or verbal rehearsal (Gattas et al., 2021). Our data are consistent with the magnitude comparison. Specifically, in the large distance condition, we observed a “first slowest” pattern, which mirrors the behavioral pattern seen in mixed trials where a magnitude comparison strategy is employed. This alignment suggests that large distance sequences, like mixed sequences, rely on magnitude comparison strategies rather than verbal rehearsal.

The absence of the RDE in the middle repetition condition can be attributed to differential interference. Specifically, repetitions at the middle position disrupt the inter-item associations crucial for small-distance processing, significantly increasing reaction times. In contrast, large-distance processing remains relatively unaffected. This selective impairment narrows the difference between the conditions, thereby abolishing the RDE.

**Figure 2:**
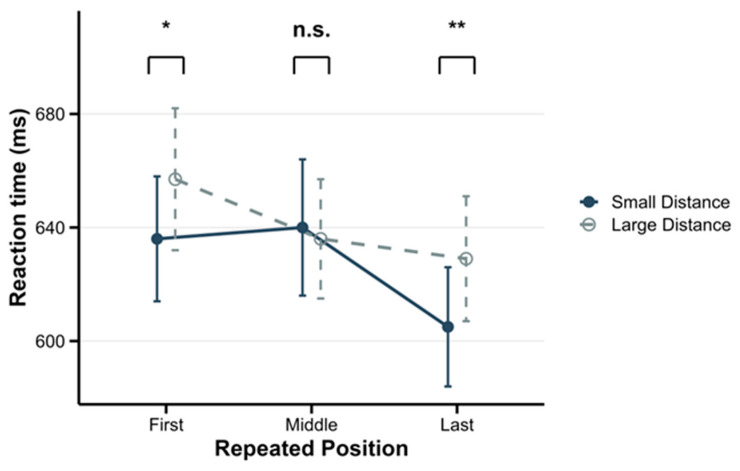
Median reaction times across ascending conditions in the numerical task. Error bars represent the within-subject standard error of the mean. * *p* < 0.05, ** *p* < 0.01, n.s. = not significant.

This interference effect, particularly for middle repetitions in small distance sequences (see Figure 2), is consistent with studies on sequence familiarity. The RDE is modulated by list composition: it can disappear when highly familiar sequences (e.g., 1-2-3) are absent (Brunner et al., 2024; Vos et al., 2021; Devlin et al., 2025). Moreover, some non-consecutive sequences (e.g., 2-4-6) can be more familiar than certain consecutive ones, indicating that overall sequence familiarity affects the RDE (Devlin et al., 2024). Consistently, the observed interference may reflect disruption of list familiarity: inserting a repeated item in a consecutive sequence (e.g., 1-2-2-3) breaks the original familiar sequence (1-2-3), slowing responses.

In addition, the Ranschburg inhibition effect in short-term memory sequences is typically most pronounced in the middle of a sequence and is often reduced or absent at the beginning and end. This pattern may provide a useful analogy for the processing of small distance sequences. Jahnke (1969) proposed that repetitions in the middle of a sequence are less distinctive, making it difficult for the brain to encode them as a single chunk (e.g., 2-2 as “2”), which hinders memory retrieval. A similar mechanism may also occur in small distance sequences in ordinal judgment tasks, leading to greater interference when middle repetitions appear.

Third, consistent with prior findings, all mixed sequences exhibited a Canonical Distance Effect, suggesting the involvement of a magnitude comparison strategy for mixed order judgments. Furthermore, mixed sequences displayed a “first repetition interference effect” (First > Middle = Last). This pattern indicates that mixed order processing relies on multiple magnitude comparisons and information integration (Lyons & Beilock, 2009; Vos et al., 2017, 2021). Specifically, the “first slowest” pattern shows that the first item is crucial for starting the comparison process. Repetitions at the first position likely hinder the initiation of magnitude comparisons and impair the integration of sequence information, resulting in slower responses.

In conclusion, Experiment 1 revealed that repetition position modulates the RDE. A significant RDE was observed in the first and last repetition conditions, but was absent in the middle repetition condition. These findings support the Item Association Account.

## 3. Experiment 2

The results of Experiment 1 provide support for the Item Association Account of the RDE. Building on these findings, Experiment 2 extended the experimental paradigm to repeated letter sequences (e.g., A-B-B-C) to investigate whether the RDE in non-strict sequences generalizes across domains.

### 3.1. Method

#### 3.1.1. Participants

A separate group of 30 participants (24 females; *M* = 22.10 years and *SD* = 1.82) was recruited for Experiment 2. Participants’ characteristics and the recruitment procedures were identical to those in Experiment 1.

#### 3.1.2. Procedure

Experiment 2 was performed in a sound-attenuated chamber. The experiment was programmed using PsychoPy software (Version 2022.1.2; Peirce et al., 2019). Visual stimuli were displayed on a 27-inch LG monitor (resolution: 2560 × 1440 pixels; refresh rate: 120 Hz) at a viewing distance of approximately 60 cm. Target letters subtended 8.2° × 1.4° of visual angle (width × height).

#### 3.1.3. Letter Order Judgment Task

In Experiment 2, participants performed a letter order judgment task. The stimuli consisted of the first nine letters of the alphabet (A–I), replacing the numbers (1–9) used in Experiment 1. All other experimental conditions were identical to those in Experiment 1.

### 3.2. Results

The overall accuracy for the order judgment task approached a ceiling effect (*M* = 92.37%; *SD* = 4.25). Given the ceiling effect in accuracy and the absence of a significant speed–accuracy trade-off (Pearson correlations: *r* = −0.229; *p* = 0.223), error trials (7.63%) and outlier trials (0.60%; defined as >3 SD from the mean) were excluded from the analysis. After excluding extreme values, the reaction time data still exhibited moderate positive skewness (skewness = 0.87) and leptokurtosis (kurtosis = 3.06). Following previous research (Devlin et al., 2024; Vogel et al., 2021; Vos et al., 2017), the median reaction time was used as the statistical measure. Table 3 presents the mean RTs and standard deviations for each condition in Experiment 2.

A 2 × 2 × 3 repeated measures ANOVA was conducted on median reaction times, with Order (ascending vs. mixed), Distance (small vs. large), and Repetition Position (first, middle, and last) as the within-subject factors. Post hoc pairwise comparisons were computed using estimated marginal means derived from the fitted model, with *p*-values adjusted using the Tukey method.

The ANOVA results revealed a significant main effect of Order: *F*(1, 29) = 22.76, *p* < 0.001, and *η_p_*^2^ = 0.44. Specifically, responses were significantly faster in the mixed condition (*M* = 1096 ms; *SD* = 268 ms) compared to the ascending condition (*M* = 1212 ms; *SD* = 302 ms). The main effect of Distance was not significant: *F*(1, 29) = 2.36, *p* = 0.136, and *η_p_*^2^ = 0.08. The main effect of Repetition Position was also not significant: *F*(2, 58) = 0.59, *p* = 0.559, and *η_p_*^2^ = 0.02.

Regarding interaction effects, a significant interaction emerged between Order and Distance, *F*(1, 29) = 123.88, *p* < 0.001, and *η_p_*^2^ = 0.81, as well as between Order and Repetition Position, *F*(2, 58) = 4.13, *p* = 0.021, and *η_p_*^2^ = 0.12. The interaction between Distance and Repetition Position was not significant: *F*(2, 58) = 1.87, *p* = 0.164, and *η_p_*^2^ = 0.06. The three-way interaction among Order, Distance, and Repetition Position was not significant: *F*(2, 58) = 2.83, *p* = 0.067, and *η_p_*^2^ = 0.09. Due to the non-significant three-way interaction, we conducted separate simple effects analyses to further examine the interactions between Order and Distance and between Order and Repetition Position.

**Figure 3:**
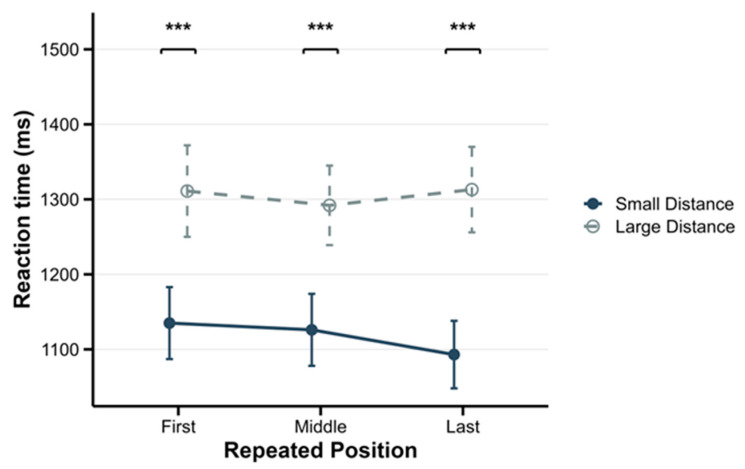
Median reaction times across ascending conditions in the letter task. Error bars represent the within-subject standard error of the mean. *** *p* < 0.001.

To decompose the significant interaction between Order and Distance, simple effects analyses were conducted. For ascending sequences, the results revealed a significant simple effect of Distance: *F*(1, 29) = 61.31, *p* < 0.001, and *η_p_*^2^ = 0.68. The reaction times for small distance sequences (*M* = 1119 ms; *SD* = 260 ms) were significantly shorter than those for large distance sequences (*M* = 1306 ms; *SD* = 313 ms), confirming a Reverse Distance Effect. See Figure 3 for the RDE patterns across repetition conditions. In the mixed condition, a significant simple effect of Distance was observed, with *F*(1, 29) = 68.02, *p* < 0.001, and *η_p_*^2^ = 0.70. Reaction times were significantly longer for small distance sequences (*M* = 1167 ms, *SD* = 262 ms) compared to large distance sequences (*M* = 1025 ms, *SD* = 255 ms), reflecting a Canonical distance effect.

To decompose the significant interaction between Order and Repetition position, simple effects analyses were conducted. In the ascending condition, the simple effect of Repetition Position was not significant: *F*(2, 28) = 0.59, *p* = 0.568, and *η_p_*^2^ = 0.04. However, in the mixed condition, the simple effect of repetition position was marginally significant, with *F*(2, 28) = 2.987, *p* = 0.067, and *η_p_*^2^ = 0.18. Post hoc comparisons revealed that responses to first position repetitions (*M* = 1111 ms; *SD* = 269 ms) were significantly slower than those to last position repetitions (*M* = 1069 ms, *SD* = 265 ms, and *p* = 0.048). No other pairwise comparisons were significant.

### 3.3. Discussion

Experiment 2 aimed to investigate the RDE in repeated letter sequences and to examine the effect of repetition position on ordinal processing.

First, regarding the effect of distance, the results indicated a significant RDE across all repetition position conditions in ascending sequences. In contrast, a significant Canonical Distance Effect (CDE) was observed across all conditions in mixed sequences.

Second, regarding repetition position, the results showed no significant differences in reaction times (RTs) across conditions for ascending sequences. In mixed sequences, although the overall pattern mirrored the numerical pattern observed in Experiment 1 (i.e., RTs were numerically longest at the first position), post hoc comparisons revealed that only the difference between the first and last positions reached statistical significance. This suggests that the magnitude comparison mechanism recruited during letter processing is less robust than that involved in numerical processing.

The persistence of the RDE and the absence of a repetition position effect in Experiment 2 align with the predictions of the Serial Scan Theory. Regarding the processing mechanisms of letter sequences, previous studies have offered different explanations. Attout et al. (2022) proposed that letter sequences primarily rely on a serial scanning mechanism. Unlike overlearned numbers, letters have lower familiarity, so ordinal information must be re-extracted via serial scanning. Their fMRI study showed that, in letter tasks, ordinal distance reliably predicted fronto-parietal cortical activity, whereas this was not the case for number tasks. Vogel et al. (2017) also supported the view that the RDE in letter sequences is driven by serial scanning. In contrast, Vos et al. (2017) argued that the RDE in letter sequences is driven by the item association mechanism. Based on Item Association and Serial Scanning Theories, they predicted that if the RDE is mainly driven by item associations, the RDE should be larger for ascending sequences, which are more familiar and strongly associated, than for descending sequences. Their results showed that the RDE was indeed larger for ascending letter sequences, and the RDE pattern for letters matched that of numbers, supporting the item association mechanism explanation.

Overall, the literature can be summarized along two lines of reasoning: first, testing experimental data against theoretical predictions (item association vs. serial scanning); second, comparing whether the RDE patterns for letters and numbers are consistent. In the present study, the RDE in repeated letter sequences was observed at all repetition positions, which aligns with predictions of the serial scanning mechanism but not with those of the item association mechanism. Moreover, the RDE patterns differed between letters and numbers: the RDE for four-item number sequences disappeared in the middle repetition condition, whereas four-item letter sequences maintained the RDE. Therefore, the ordinal processing of repeated letter sequences is primarily driven by the serial scanning mechanism.

The discrepancy between the present study and Vos et al. (2017) may be attributed to differences in experimental materials. A primary distinction lies in the experimental materials: this study employed four-item repeated letter sequences, whereas Vos et al. (2017) used standard letter triplets. The increased sequence length and the interference from repetitions likely elevated processing demands, biasing participants toward a serial scanning strategy; conversely, shorter and simpler triplets are more likely to facilitate associative strategies.

In summary, despite the robust persistence of the effect, repetition position did not significantly modulate the RDE for letters, contrary to the findings for numbers. This pattern aligns with the predictions of the Serial Scanning Account.

## 4. General Discussion

The present study investigated the ordinal processing of repeated item sequences to reveal their underlying mechanisms, clarify the generalizability of the Reverse Distance Effect (RDE) across different sequence types. Experiment 1’s results indicate that in numerical sequences, repeated numbers significantly modulated the RDE: the RDE disappeared in the middle repetition condition but remained in the first and last repetition conditions. In contrast, Experiment 2 revealed that repeated letters did not modulate the RDE pattern; the effect remained robust across conditions, with the repetition position modulating reaction times exclusively in the mixed order condition. Taken together, these findings demonstrate that the RDE generalizes across sequence types, yet it may diminish or disappear when the middle repetition condition induces differential interference.

The main findings of this study can be summarized in three key aspects: the persistence of the RDE in ordered sequences, the canonical distance effect observed in mixed sequences, and the contrasting patterns of ordinal processing across numbers and letters.

First, the combined results of Experiments 1 and 2 indicate that, overall, similarly to non-repeated sequences, the RDE generalizes across different sequence types. However, the effect vanishes when repeated items exert differential interference on small and large distance sequences. Specifically, for numerical sequences, an RDE was observed in the first and last repetition conditions, whereas the middle repetition condition yielded neither an RDE nor a canonical distance effect. This pattern can be explained by the Item Association view, which posits that small and large distances rely on distinct processing mechanisms (Devlin et al., 2022; Vos et al., 2017). As a result, the position of repeated items produces differential interference on small- and large distance sequences. In the first repetition condition, both small and large distance sequences suffered significant interference; in the middle condition, interference was severe for small distance sequences but negligible for large distance ones; and in the last condition, interference was minimal for both.

In contrast, for letter sequences, a robust RDE was observed across all repetition positions, as the magnitude of interference remained comparable regardless of position. This interference pattern aligns with the Serial Scanning prediction. As repeated items do not alter the spatial scanning distance along the mental number line, the degree of interference is comparable across all positions (Dehaene et al., 1993; Dewulf et al., 2024; Franklin et al., 2009; Turconi et al., 2006). In summary, it appears that the RDE persists when the degree of interference is comparable between small and large distance sequences, but vanishes when differential interference occurs, particularly when interference is disproportionately greater for small distance sequences.

Second, in mixed sequences, both numbers and letters exhibited a significant Canonical Distance Effect. Crucially, the response times for the first repetition condition were significantly longer than those for the middle and last repetition conditions. This pattern suggests that the Canonical Distance Effect in mixed sequences is not solely influenced by representational overlap (Vos et al., 2021; Vogel et al., 2021) but also relies heavily on the continuity of multiple comparisons and relational integration. Responses were slowest in the first repetition condition, implying that the initiation of magnitude comparison plays a vital role in the processing of mixed sequences, as a repetition at the first position disrupts the construction of a coherent relational structure. In letter sequences, this interference effect was similar to that observed for numbers, but weaker. This aligns with previous studies suggesting that letters and numbers engage similar ordinal processing mechanisms (Fias et al., 2007; Michalewski et al., 1988).

Third, the presence of the RDE in the letter task indicates that this effect extends to non-numerical materials. The different RDE patterns observed for numbers (Experiment 1) and letters (Experiment 2) suggest that ordinal processing may rely on different mechanisms across materials domains. Specifically, numerical sequences are more likely to engage an item association mechanism (Vos et al., 2017), which is sensitive to disruptions caused by repeated items. In contrast, letter sequences appear to rely more on a serial scanning mechanism (Franklin et al., 2009), in which repeated items do not alter the overall scanning distance and therefore produce less interference.

Although differences in RDE mechanisms across materials may suggest domain-specific ordinal coding, caution is necessary. In our study, numbers and letters exhibited similar canonical distance effects in unfamiliar mixed sequences (Vos et al., 2021), but differed in ordered sequences. One possible explanation is familiarity: familiarity appears to influence not only behavioral outcomes but also the underlying mechanisms of ordinal processing. In ordered sequences, familiar sequences tend to engage item-association processing, whereas unfamiliar sequences rely on serial scanning (Lyons et al., 2014). In mixed sequences, the sequence structures are infrequent and unfamiliar, making spatial scanning difficult; therefore, participants primarily rely on magnitude comparison strategies (Vos et al., 2021). An alternative explanation is domain-specific ordinal coding. However, stable domain-specific coding would predict consistent differences across sequence types, which were not observed; instead, the pattern of our results is more consistent with predictions based on familiarity. Thus, we suggest that the differences in ordinal processing may be largely driven by familiarity.

Current evidence remains insufficient to determine whether ordinal coding is domain general or domain specific. Attout et al. (2022) found that fronto-parietal activity predicted letters but not numbers, yet this activity was also associated with a non-ordinal brightness task, suggesting reduced attentional demands for overlearned numbers rather than true domain-specific coding. Vos et al. (2017) reported similar mechanisms for numbers and letters, but it remains unclear whether this reflects general learning associations or domain-general ordinal coding. Taken together, regardless of similarities or differences in behavioral mechanisms and neural evidence, existing findings provide little decisive support for domain-general versus domain-specific ordinal coding.

Familiarity is a critical factor in assessing domain-general versus domain-specific ordinal coding. To date, only one study (Devlin et al., 2024) has directly assessed subjective sequence familiarity. Because familiarity influences processing mechanisms, it leads to covariance of mechanisms, which complicates interpretation. Future studies should manipulate familiarity and frequency using artificial symbol paradigms to achieve a double dissociation and more directly test domain-general versus domain-specific ordinal coding.

Future research should systematically compare the processing mechanisms of repeated and non-repeated item sequences, particularly focusing on the origin of the RDE. A recent systematic review by Harju and Van Hoof (2025) highlights the methodological diversity in measuring ordinality. This suggests that the processing of repeated item sequences would benefit from being examined using multiple measurement approaches. Additionally, an interesting possibility is that middle repetitions may also serve as a cognitive anchor in other contexts (e.g., 1|22|3). Similarly to number 5 in the 1–9 numeric range (Pinto et al., 2019), this middle repetition divides the sequence into two chunks and may guide inter-item association strategies. Finally, future research should consider the effects of cultural familiarity on ordinal processing. Examining ordinal processing across different cultural or linguistic contexts can help clarify whether it is influenced by symbol-specific experience.

In conclusion, this study firstly investigated the ordinal processing of repeated numerical and letter sequences. Overall, the RDE generalizes to repeated item sequences; however, the effect may diminish when repeated items induce differential interference between sequences of small and large distances. Furthermore, in mixed sequences, the canonical distance effect was observed, consistent with non-repeated sequences. Finally, the presence of the RDE in the letter task shows that this effect extends to non-numerical materials. The different interference patterns caused by repetition position suggest that processing repeated item sequences involves multiple mechanisms.

## Figures and Tables

**Table 1:** Predicted outcomes in ordered sequences of different theoretical accounts of the RDE.

Theoretical Account	Prediction for the RDE	Interaction Between Distance and Repetition Position
Item Association Account	Present but variable; modulated by the degree of interference induced by repetition position.	Interaction present: stronger interference reduces or eliminates the RDE, whereas weaker interference preserves it.
Serial Scanning Account	Robust RDE; the RDE is unaffected by repetition.	No interaction: the RDE remains stable across repetition positions.
Magnitude Comparison Account	No RDE; instead, a canonical distance effect is expected.	——
Distinct Processing Hypothesis	No distance-related effects (neither RDE nor CDE) are expected under any conditions	No interaction; processing is independent of distance.

**Table 2:** The mean and standard deviation of the median reaction times (in ms) across all conditions in Experiment 1.

	Ascending			Mixed		
	First	Middle	Last	First	Middle	Last
Small distance	636 ± 128	640 ± 135	605 ± 117	725 ± 139	705 ± 133	705 ± 150
Large distance	657 ± 143	636 ± 120	629 ± 124	686 ± 145	651 ± 130	634 ± 113

**Table 3:** The mean and standard deviation of the median reaction times (in ms) across all conditions in Experiment 2.

	Ascending			Mixed		
	First	Middle	Last	First	Middle	Last
Small distance	1135 ± 266	1126 ± 267	1093 ± 251	1162 ± 260	1178 ± 274	1158 ± 261
Large distance	1311 ± 337	1292 ± 291	1313 ± 317	1060 ± 273	1033 ± 251	980 ± 241

## Data Availability

The original data presented in the study are openly available in OSF https://osf.io/swc7t/ (accessed on 15 January 2026).

## Supplementary Materials

The following supporting information can be downloaded at: https://www.mdpi.com/article/10.3390/bs16040582/s1, Table S1. Complete trial list for the numerical order judgment task in Experiment 1. Table S2. Complete trial list for the letter order judgment task in Experiment 2.

## Abbreviations

The following abbreviations are used in this manuscript:

RDE	Reverse Distance Effect
CDE	Canonical Distance Effect

## References

[B1-behavsci-16-00582] Attout L., Leroy N., Majerus S. (2022). The neural representation of ordinal information: Domain-specific or domain-general?. Cerebral Cortex.

[B2-behavsci-16-00582] Brunner C., Schadenbauer P., Schröder N., Grabner R. H., Vogel S. E. (2024). Electrophysiological correlates of symbolic numerical order processing. PLoS ONE.

[B3-behavsci-16-00582] Crowder R. G. (1968). Intraserial repetition effects in immediate memory. Journal of Verbal Learning and Verbal Behavior.

[B4-behavsci-16-00582] Dehaene S., Akhavein R. (1995). Attention, automaticity, and levels of representation in number processing. Journal of Experimental Psychology: Learning, Memory, and Cognition.

[B5-behavsci-16-00582] Dehaene S., Bossini S., Giraux P. (1993). The mental representation of parity and number magnitude. Journal of Experimental Psychology: General.

[B6-behavsci-16-00582] Devlin D., Moeller K., Reynvoet B., Sella F. (2022). A critical review of number order judgements and arithmetic: What do order verification tasks actually measure?. Cognitive Development.

[B7-behavsci-16-00582] Devlin D., Moeller K., Xenidou-Dervou I., Reynvoet B., Sella F. (2024). Familiar sequences are processed faster than unfamiliar sequences, even when they do not match the count-list. Cognitive Science.

[B8-behavsci-16-00582] Devlin D., Moeller K., Xenidou-Dervou I., Reynvoet B., Sella F. (2025). The presence of the reverse distance effect depends on the familiarity of the sequences being processed. Psychological Research.

[B9-behavsci-16-00582] Dewulf M., Gevers W., Antoine S. (2024). The ordinal distance effect in working memory: Does it exist in the absence of confounds?. Psychological Research.

[B10-behavsci-16-00582] Dubinkina N., Sella F., Reynvoet B. (2021). Symbolic number ordering and its underlying strategies examined through self-reports. Journal of Cognition.

[B11-behavsci-16-00582] Duncan G. J., Dowsett C. J., Claessens A., Magnuson K., Huston A. C., Klebanov P., Pagani L. S., Feinstein L., Engel M., Brooks-Gunn J., Sexton H., Duckworth K., Japel C. (2007). School readiness and later achievement. Developmental Psychology.

[B12-behavsci-16-00582] Fias W., Lammertyn J., Caessens B., Orban G. A. (2007). Processing of abstract ordinal knowledge in the horizontal segment of the intraparietal sulcus. The Journal of Neuroscience.

[B13-behavsci-16-00582] Franklin M. S., Jonides J., Smith E. E. (2009). Processing of order information for numbers and months. Memory & Cognition.

[B14-behavsci-16-00582] Gattas S. U., Bugden S., Lyons I. M. (2021). Rules of order: Evidence for a novel influence on ordinal processing of numbers. Journal of Experimental Psychology: General.

[B15-behavsci-16-00582] Goffin C., Ansari D. (2016). Beyond magnitude: Judging ordinality of symbolic number is unrelated to magnitude comparison and independently relates to individual differences in arithmetic. Cognition.

[B16-behavsci-16-00582] Harju H., Van Hoof J. (2025). Putting things in order! A systematic literature review on the ambiguity in descriptions, measurement, and terminology of ordinality. PsyArXiv.

[B17-behavsci-16-00582] Jahnke J. C. (1969). The Ranschburg effect. Psychological Review.

[B18-behavsci-16-00582] Johnson A. J., Skinner R., Takwoingi P., Miles C. (2019). Tactile memory Ranschburg effects under conditions of concurrent articulation. Quarterly Journal of Experimental Psychology.

[B19-behavsci-16-00582] Lyons I. M., Beilock S. L. (2009). Beyond quantity: Individual differences in working memory and the ordinal understanding of numerical symbols. Cognition.

[B20-behavsci-16-00582] Lyons I. M., Beilock S. L. (2013). Ordinality and the nature of symbolic numbers. The Journal of Neuroscience.

[B21-behavsci-16-00582] Lyons I. M., Price G. R., Vaessen A., Blomert L., Ansari D. (2014). Numerical predictors of arithmetic success in grades 1–6. Developmental Science.

[B22-behavsci-16-00582] Lyons I. M., Vogel S. E., Ansari D. (2016). On the ordinality of numbers. Progress in brain research.

[B23-behavsci-16-00582] Michalewski H. J., Kamel A.-W. M., Starr A. (1988). Brain potentials during mental distance judgments. International Journal of Psychophysiology.

[B24-behavsci-16-00582] Morsanyi K., O’Mahony E., McCormack T. (2017). Number comparison and number ordering as predictors of arithmetic performance in adults: Exploring the link between the two skills, and investigating the question of domain-specificity. Quarterly Journal of Experimental Psychology.

[B25-behavsci-16-00582] Moyer R. S., Landauer T. K. (1967). Time required for judgements of numerical inequality. Nature.

[B26-behavsci-16-00582] Peirce J., Gray J. R., Simpson S., MacAskill M., Höchenberger R., Sogo H., Kastman E., Lindeløv J. K. (2019). PsychoPy2: Experiments in behavior made easy. Behavior Research Methods.

[B27-behavsci-16-00582] Pinto M., Pellegrino M., Lasaponara S., Cestari V., Doricchi F. (2019). Contrasting left/right codes for response selection must not be necessarily associated with contrasting numerical features to get the SNARC. Acta Psychologica.

[B28-behavsci-16-00582] Ranschburg P. (1905). Über die bedeutung der ähnlichkeit beim erlernen, behalten, und bei der reproduktion. Journal der Psychologie und Neurologie.

[B29-behavsci-16-00582] R Core Team (2023). R: A language and environment for statistical computing *(Version 4.3.2) [Computer software]*.

[B30-behavsci-16-00582] Ritchie S. J., Bates T. C. (2013). Enduring links from childhood mathematics and reading achievement to adult socioeconomic status. Psychological Science.

[B31-behavsci-16-00582] Rosen K. H., Krithivasan K. (2013). Discrete mathematics and its applications.

[B32-behavsci-16-00582] RStudio Team (2022). RStudio: Integrated development environment for R *[Computer software]*.

[B33-behavsci-16-00582] Sasanguie D., Lyons I. M., De Smedt B., Reynvoet B. (2017). Unpacking symbolic number comparison and its relation with arithmetic in adults. Cognition.

[B34-behavsci-16-00582] Sella F., Sasanguie D., Reynvoet B. (2020). Judging the order of numbers relies on familiarity rather than activating the mental number line. Acta Psychologica.

[B35-behavsci-16-00582] Serra M., Nairne J. S. (2000). Part—Set cuing of order information: Implications for associative theories of serial order memory. Memory & Cognition.

[B36-behavsci-16-00582] Turconi E., Campbell J. I. D., Seron X. (2006). Numerical order and quantity processing in number comparison. Cognition.

[B37-behavsci-16-00582] van Opstal F., Verguts T. (2011). The origins of the numerical distance effect: The same–different task. Journal of Cognitive Psychology.

[B38-behavsci-16-00582] Verguts T., Van Opstal F. (2005). Dissociation of the distance effect and size effect in one-digit numbers. Psychonomic Bulletin & Review.

[B39-behavsci-16-00582] Vogel S. E., Faulkenberry T. J., Grabner R. H. (2021). Quantitative and qualitative differences in the canonical and the reverse distance effect and their selective association with arithmetic and mathematical competencies. Frontiers in Education.

[B40-behavsci-16-00582] Vogel S. E., Haigh T., Sommerauer G., Spindler M., Brunner C., Lyons I. M., Grabner R. H. (2017). Processing the order of symbolic numbers: A reliable and unique predictor of arithmetic fluency. Journal of Numerical Cognition.

[B41-behavsci-16-00582] Vogel S. E., Remark A., Ansari D. (2015). Differential processing of symbolic numerical magnitude and order in first-grade children. Journal of Experimental Child Psychology.

[B42-behavsci-16-00582] Vos H., Gevers W., Reynvoet B., Xenidou-Dervou I. (2021). Ordinality: The importance of its trial list composition and examining its relation with adults’ arithmetic and mathematical reasoning. Quarterly Journal of Experimental Psychology.

[B43-behavsci-16-00582] Vos H., Sasanguie D., Gevers W., Reynvoet B. (2017). The role of general and number-specific order processing in adults’ arithmetic performance. Journal of Cognitive Psychology.

[B44-behavsci-16-00582] Wong B., Bull R., Ansari D. (2018). Magnitude processing of written number words is influenced by task, rather than notation. Acta Psychologica.

[B45-behavsci-16-00582] Wong B., Bull R., Ansari D., Watson D. M., Liem G. A. D. (2022). Order processing of number symbols is influenced by direction, but not format. Quarterly Journal of Experimental Psychology.

