# Supplementary material for: The Reverse Distance Effect in Ordinal Processing of Repeated Item Sequences
Source: Behav Sci (Basel). 2026 Apr 13;16(4):582. doi: 10.3390/bs16040582 (PMC13112960; doi:10.3390/bs16040582)
Supplement: Supplementary file 1 [file behavsci-16-00582-s001.zip › behavsci-4107085-supplementary.pdf]

Table S1. Complete trial list for the numerical order judgment task in Experiment 1.

	Ascending sequence			Mixed sequences		
	First repeated	Middle repeated	Last repeated	First repeated	Middle repeated	Last repeated
small distance	1123	1223	1233	2231	2331	2311
	2234	2334	2344	3342	3442	3422
	3345	3445	3455	4453	4553	4533
	4456	4556	4566	5564	5664	5644
	5567	5667	5677	6675	6775	6755
	6678	6778	6788	7786	7886	7866
	7789	7889	7899	8897	8997	8977
large distance	1135	1335	1355	3351	3551	3511
	2246	2446	2466	4462	4662	4622
	4468	4668	4688	6684	6884	6844
	5579	5779	5799	7795	7995	7955
	1147	1447	1477	4471	4771	4711
	2258	2558	2588	5582	5882	5822
	3369	3669	3699	6693	6993	6933

Table S2. Complete trial list for the letter order judgment task in Experiment 2.

	Ascending sequence			Mixed sequences		
	First repeated	Middle repeated	Last repeated	First repeated	Middle repeated	Last repeated
small distance	AABC	ABBC	ABCC	BBCA	BCCA	BCAA
	BBCD	BCCD	BCDD	CCDB	CDDB	CDBB
	CCDE	CDDE	CDEE	DDEC	DEEC	DECC
	DDEF	DEEF	DEFF	EEFD	EFFD	EFDD
	EEFF	EFFF	EFFF	FFFE	FFFE	FFEE
	FFFH	FFFH	FFHH	FFHF	FHHF	FHFF
	FFHI	FHHI	FHII	HHIF	HIIF	HIFF
large distance	AACE	ACCE	ACEE	CCEA	CEEA	CEAA
	BBDF	BDDF	BDFF	DDFB	DFFB	DFBB
	DDFH	DFFH	DFHH	FFHD	FHHD	FHDD
	EEFI	EFFI	EFII	FFIE	FIIE	FIEE
	AADF	ADDF	ADFF	DDFA	DFFA	DFAA
	BBEH	BEEH	BEHH	EEHB	EHHB	EHBB
	CCFI	CFFI	CFII	FFIC	FIIC	FICC
